# Deep neural networks for crack detection inside structures

**DOI:** 10.1038/s41598-024-54494-y

**Published:** 2024-02-23

**Authors:** Fatahlla Moreh, Hao Lyu, Zarghaam Haider Rizvi, Frank Wuttke

**Affiliations:** 1 Geomechanics and Geotechnics, Kiel University, Kiel, 24118 Germany; 2 Competence Centre for Geo-Energy, Kiel University, Kiel, 24118 Germany; 3 GeoAnalysis Engineering GmbH, Kiel, 24118 Germany

**Keywords:** Neural network, Deep learning, Crack detection, Wavefield, Civil engineering, Computer science

## Abstract

Crack detection is a long-standing topic in structural health monitoring. Conventional damage detection techniques rely on intensive, time-consuming, resource-intensive intervention. The current trend of crack detection emphasizes using deep neural networks to build an automated pipeline from measured signals to damaged areas. This work focuses on the seismic-wave-based technique of crack detection for plate structures. Previous work proposed an encoder–decoder network to extract crack-related wave patterns from measured wave signals and predict crack existence on the plate. We extend previous work with extensive experiments on different network components and a data preprocessing strategy. The proposed methods are tested on an expanded crack detection dataset. We found that a robust backbone network, such as Densely Connected Convolutional Network (DenseNet) can effectively extract the features characterizing cracks of wave signals, and by using the reference wave field for normalization, the accuracy of detecting small cracks can be further improved.

## Introduction

Crack detection is a long-standing structural health monitoring (SHM) research topic. Cracks can change material behaviour and weaken structural strength. Even tiny cracks can evolve into severe damage to structures and cause heavy loss of property and life. The ultimate goal of SHM is to estimate the structure’s state from sensor data and use the information to make decisions regarding the maintenance and decommissioning of the structure. Rytter pointed out that the SHM task is a hierarchy of levels, including detection, localization, assessment, and prediction^1^. Recent developments in SHM show increasing demand for more sophisticated data processing and analyzing techniques for the four levels of SHM tasks^2^. Deep learning (DL) approaches are getting particular attention^3^. Because DL approaches enable machines to automatically learn task-specified features without relying on hand-crafted features from experts and researchers^4^. Deep neural networks (DNN) take raw data as input and develop hierarchical representations through stacked neural network layers with nonlinear mappings. Automated image acquisition platforms and deep learning image processing algorithms are extensively used in current research for surface crack detection, replacing manual visual inspection^5–7^. These algorithms use images as input and estimate damage from different levels, ranging from categorizing damage/non-damage classes to identifying different severity levels or damage types. Attempts were also made to use DNNs in wave propagation-based damage detection. The forms of input data greatly influence the selection of DNN variations. Dense Networks, 1D-Convolutional Neural Networks (CNN), Recurrent Neural Networks (RNN), and Long Short-Term Memory (LSTM) are used to learn vibration signatures from the time domain^8–10^. 2D CNNs are usually employed to learn damage-relevant features from the frequency domain^11^ or an array of sensors^12^. The propagation properties of waves depend on the properties of the media they travel through and the parameters of the excited wave. The interaction between waves and cracks modifies the wavefield. The wavefront reflects and refracts when it encounters the cracked interface. When assuming that sensors are used to capture this modification, a crack detection method aims to establish the connection between the observed wave data and the damaged region. In previous work, we developed a spatially asymmetric encoder-decoder model to approach the connection^13,14^. The detection of cracks on plates is addressed as a semantic segmentation problem, where each point in the material domain is classified into damaged/intact classes. The “encoder network” receives observed data as input and extracts crack-related features. The “decoder network” masks the damaged region according to the extracted features. For the specific task, the developed model is tailored to the specific challenges of the problem because it takes the wavefield measurements from a sparsely distributed sensor array and makes dense predictions for the whole material domain. The backbone network of the encoder, e.g., Visual Geometry Group (VGG)^15^ or Residual Networks^16^, is modified to a 1D version according to the format of wavefield data. Upsampling layers rescale the encoded features into a desired spatial resolution.

In this work, we extend the previous work, set up a baseline with extensive experiments on different components of the asymmetric encoder-decoder structure, and propose a new normalization strategy for input wave signals. The rest of the paper is organized as follows: Sect. ”Related work” briefly introduces recent crack detection achievements and highlights the deep learning approaches. Section Problem definition presents the formal definition of our crack detection problem and indicates the difference between the crack detection problem and the image semantic segmentation problem. The crack detection dataset is reported in the section “The crack detection dataset”. In “Deep neural networks for crack detection” and “Benchmarking experiments”, we have described the DNN models and reported the results of benchmarking experiments. Finally, in the “Conclusion”, we discussed the paper with final concerns and remarks.

## Related work

### Damage detection in shell structures

Active lamb wave-based methods are one of the most promising techniques for SHM of shell structures. Lamb waves can travel long distances without significant loss in intensity. Thus, large areas of shell structures can be examined with a sparse array of transducers. Yuan et al. developed an active monitoring system for honeycomb sandwich and carbon fibre composite structures based on wide-band Lamb waves^17^. They employed a two-layer neural network to extract the wave signature of two types of damage: delamination and impact damage. Golato et al. developed the SHM method based on the sparse reconstruction technique to identify the size and shape of the defect^18^. The proposed method produces a heat map of damage probability for the region of interest through the fundamental $A_0$ wave mode propagation captured by multiple sensors. Their method was validated using simulated data for a thin aluminium plate. The interaction of Lamb wave modes with damages can be strongly dispersed, which leads to complex signals being interpreted. Numerical simulations are often needed to offer some possibilities to interpret measured signals. Gravenkamp and his colleagues applied the scaled boundary finite element method (SBFEM) to simulate Lamb waves in cracked plates with more efficiency and accuracy compared to FEM^19^. Besides sparsely distributed sensors, complete wavefield data is also an effective damage detection, localization, and visualization tool. Kudela et al. applied a set of signal processing techniques to extract crack-relevant features without using baseline signals^20^. A high-resolution scanning laser Doppler vibrometer acquires the complete wavefield data. However, applying the methods to high-resolution wavefield data is still challenging, requiring an extended processing time and much storage space.

Another category of SHM systems passively receives the sound waves from a cracking site in a structure, namely the acoustic emission (AE). AEs are caused by sudden changes of strain in fracturing material that lead to a burst of energy released by high-frequency sound waves. For the general application of AE in SHM, we refer to the review paper^21^. Here, we only name several AE-based SHM approaches applied to shell structures. Farhidzadeh and his colleagues analyzed the b-value of AEs to monitor the fracture process of a large-scale reinforced concrete shear wall^22^. The b-value is the log-linear slope of the frequency-magnitude distribution of acoustic emissions. The fracturing events are distinguished from baseline data through a statistical outlier analysis. The authors have identified the pattern of fracturing in AE signals. These patterns, though not used to indicate the location of the crack, can be used as an early warning of structural failure. Van Driessche et al. investigated the AE patterns in a fracturing wall of textile-reinforced cement for two types of damages: mode-I cracking and shear-dominated debonding and delamination^23^. Argus et al. developed an AE-based SHM system for thin-walled carbon fibre reinforced plastics (CFRP) structure^24^. The AEs were simulated through an artificial source and received by piezoelectric sensors. They employed an artificial neural network to localize the sound source from the monitored acoustic signals with high precision.

#### Deep learning approaches for crack detection

The deep learning approaches for crack detection can be classified into two types according to their input data, i.e., the vision-based approach and the wave-based approach.

The vision-based approach uses images and videos as input and adopts the advanced DNNs, which were initially designed for computer vision tasks for surface inspection. Depending on the granularity of the crack detection task, vision-based methods can be categorized into three types: (1). image classification, i.e., categorizing image patches into “damaged” or “intact” classes or into different severity levels^5,25,26^, (2). object detection or region-proposal methods, identifying the crack’s existence and predicting its bounding box on the image patch^7,27,28^, and (3). semantic segmentation methods, classifying each pixel of an image patch into “damage” or “non-damage” classes^6,29–34^. The semantic segmentation approaches have been widely applied as an alternative to visual inspection for monitoring civil structures, such as roads^35^, tunnels^36^, and bridges^37^.

The wave-based approach is based on the idea that the crack’s existence changes the physical properties of the structure (e.g., mass, damping, and stiffness) and thus leads to the change of modal parameters (e.g., natural frequencies, modal shapes, modal damping)^38^. Usually, wave propagation can be induced by natural sources (e.g., seismic) or human-made sources (e.g., guided waves). The measured responses, such as displacement, velocity, and acceleration, can be compared with the responses of a predefined numeric model or the benchmark responses from the non-damage structure or analyzed statistically without any benchmark model.

Recent studies demonstrate that DNN can be a powerful tool to extract wave patterns from wave response signals even with high noise levels^39,40^. Abdeljaber and his colleagues presented a 1D-CNN model to identify and localize structural damage from raw vibration signals^41^. A network of accelerometers acquires these signals, and the damage is simulated as a loose bolt in the structure. For each damage case, a separate CNN is optimized to estimate the probability of damage (PoD). The model is evaluated for undamaged, single, and multiple damage cases with high accuracy (0.54% average error). In the following paper, they applied 1D-CNN to address the binary damage detection problem, distinguishing between undamaged and damaged cases^8^. Based on simulated vibration responses, Lin et al. demonstrate that a 1D-CNN model performs better than the signal processing method that analyses the wavelet packet transform (WPT) component energy to identify a local change in the system parameters^42^. The Neural Network (NN) model is robust to white Gaussian noise and can detect the location of single/multiple damages with corresponding damage degrees. To generate vibration responses, the authors build a supported beam model consisting of 10 identical Euler-Bernoulli beam elements and calculate the vertical accelerations of each beam element under different damage locations, damage levels, and burst random excitation. Besides 1D-CNN, Long-Short Term Memory (LSTM) network is also commonly used as the pattern extractor for damage detection. Hung et al. build an LSTM-based single end-to-end network that directly takes raw acceleration time-series without requiring any signal preprocessing step^10^. The proposed approach is evaluated on the vibration data collected from a laboratory-scale three-story frame and produces accurate damage detection results. Considering the difficulty of collecting training data with precisely labelled damages from the in-service infrastructures, Wang and Cha proposed an unsupervised method that combines CNN and support vector machine (SVM) to learn signal patterns from a known undamaged scenario and identify damages of unknown scenarios^43^. Though response data can be easily generated from numeric simulations, collecting data for every structure part is impractical.

Goh et al. represent a study on predicting unmeasured mode shape magnitudes based on a limited number of measured data with the NN model^44^. Compared with the Cubic Spline interpolation (CS) method, the NN model provides more reliable results with limited measurement points. Ding et al. present a sparse deep belief network (DBN) for structural damage detection with a noisy and limited amount of data^45^. The proposed model takes vibration characteristics in the modal domain, i.e., natural frequencies and mode shapes, as input to the network and predicts the structure’s damage locations and levels. To consider the sensors’ geometric location, Sajedi and Liang developed a grid-based methodology for damage segmentation in large-scale civil infrastructures^46^. Their fully convolutional encoder-decoder neural network takes wave response from an array of sensors and predicts damage status for each sensor location. Cumulative intensity measures train the model as the input and damage states of nodes as output. Their proposed approach yielded global accuracies of 96.3% and 93.2% for detecting damage location and severity in an FE model, respectively. This grid-based methodology is very similar to the proposed model in this paper. One of the significant differences is that our model takes wave responses from sparsely distributed sensors as input and produces very dense predictions.

## Problem definition

The crack detection problem of a solid structure can be formalized as follows: Given a solid structure represented by a physical domain $\Omega \subset {\mathbb {R}}^n$ ($n=2,3$), an internal crack can be written as a function $\phi (\varvec{x})=1$, where $\varvec{x} \in {\mathscr {R}}^n$ is a point in the domain. The intact area is represented as $\phi (\varvec{x})=0$. Given *N* sensors at fixed locations ${\varvec{x}_0, \cdots , \varvec{x}_i, \cdots , \varvec{x}_N}$, the recorded wave data at time *t* of sensor location $\varvec{x}_i$ is denoted as $\varvec{u}_{(i,t)}$. For example, if the observation period is given from $t_0=0$ to $t=T$, we omit the temporal index without any ambiguity. The crack detection problem can be addressed by finding a functional ${\mathscr {G}}: f(\varvec{u}_0, \cdots , \varvec{u}_N) \mapsto \phi (\varvec{x})$.

In practice, we can use a neural network of trainable parameters $\varvec{\theta }$ to approximate the functional

1 $$\begin{aligned} \hat{{\mathscr {G}}}_{\varvec{\theta }}(\varvec{u}_0, \cdots , \varvec{u}_N)(\varvec{x}), \end{aligned}$$

Training such a neural network means finding optimal parameters $\varvec{\theta }^{*}$ that

2 $$\begin{aligned} \varvec{\theta }^{*} = \mathop {\mathrm {arg\,min}}\limits _{\varvec{\theta }}\int _{\Omega }\big \Vert \hat{{\mathscr {G}}}_{\varvec{\theta }}(\varvec{u}_0, \cdots , \varvec{u}_N)(\varvec{x}) - \phi (\varvec{x})\big \Vert \textrm{d}\varvec{x}. \end{aligned}$$

To simplify the problem, a 2D rectangular domain is assumed. The physical domain is decomposed into a regular grid tessellation. The cell size of the grid determines the spatial resolution of the decomposition. Determining cell size requires considering the size of the crack region, material properties, excitation source intensity, etc. Too small cell size increases the difficulty in prediction, while too large cell size makes it difficult to determine the exact location of the crack. Grid cell can be indexed by a row-column pair $\langle i, j \rangle$. Then, we can convert the above optimization problem to a discretized version,

3 $$\begin{aligned} \varvec{\theta }^{*} = \mathop {\mathrm {arg\,min}}\limits _{\varvec{\theta }}\sum _{i, j} \big \Vert \hat{{\mathscr {G}}}_{\varvec{\theta }}(\varvec{u}_0, \cdots , \varvec{u}_N)(\varvec{x}_{i, j}) - \phi (\varvec{x}_{i, j})\big \Vert . \end{aligned}$$

The simplified problem is comparable to the image semantic segmentation task. Semantic segmentation is a computer vision task that aims to classify each pixel of an input image into a fixed set of semantic categories without distinguishing object instances. Deciding what class a pixel belongs to requires information from the pixel itself and the context information from its neighbouring pixel. This requirement leads to a widely accepted encoder-decoder architecture in deep learning. The encoder learns highly abstracted but coarse semantic information in deep layers and fine appearance information in shallow layers^47^. The decoder network semantically projects the information learnt by the encoder (lower resolution) onto the pixel space (higher resolution) to get a dense classification. Some models also employ “skip” connections to combine shallow and deep features^47–50^. The encoder usually employs well-established CNN architectures, such as VGGNet^51^ or Residual Network (ResNet)^52^; in the decoder, the combinations of upsampling and trainable layers achieve the pixel-level prediction.

This encoder-decoder architecture has been widely used in computer vision tasks like image segmentation. Similar architectures are also applied to visually inspect surface cracks, where hand-holding devices or autonomous platforms can collect photos^34,53,54^. Image segmentation requires an understanding of the image from both semantics and location, which induces the tension between capturing global information (semantics) and capturing local information (location)^47^. Many models use the spatial correspondence between the input and output domains and add a skip connection between the deep features in the encoder network and decoder network^47,48^.

Crack detection from observed wave data is comparable to the binary semantic segmentation task, where each image is classified as damaged or intact, where each pixel of the material domain requires an assignment of the class. The model requires learning semantic and locational information from condensed wave patterns. Thus, similar ideas were borrowed from image segmentation models. However, there is an essential difference between wave-based crack detection and image semantic segmentation, i.e., the spatial mismatch between input and output. The ultimate aim of a crack detection model is to use the data from the sparsely distributed sensor array to make dense predictions for the solid domain. Though regularly distributed sensor arrays imply locational information in the data, they can not be easily related to the spatial locations of the output. The spatial dimension of sensors (sparse) is mismatched with the desired spatial dimension of crack predictions (dense). Besides, the measured wavefield also involves temporal dimension and x-/y-displacements instead of image channels. In our model, this information is condensed and combined by the encoder in a temporal-to-spatial way.

## The crack detection dataset

The wave-crack interaction dataset consists of homogeneous 2D samples of a rectangular domain. Each sample contains a randomly assigned crack (or no crack). The previous studies built a small dataset and illustrated the influence of crack size on the model’s performance^13,14^. Specifically, much smaller cracks are more difficult to detect than larger cracks. To facilitate a fair evaluation, it’s necessary to have a similar distribution of samples of different crack sizes in training, validation, and testing datasets. The previous dataset and the random selection of the validation set from the training set fail to address the concerns. In this work, we expanded the dataset size by adding more samples and re-sampling them for the three subsets. The dynamic lattice element method simulates their dynamic behaviour under an external force.

### Dynamic lattice element method

Compared to crack generation in real-world samples, numeric simulation is a fast and efficient alternative for producing massive data to meet the data requirements of training deep neural networks. On the other hand, producing a large number of real-world samples is prohibitively expansive^55^. To be compatible with the previous dataset, we adopted the same technique for simulating the dynamic behaviour of the samples, i.e., dynamic Lattice Element Method (dLEM)^56,57^. The lattice element method (LEM) consists of mesoscale models that decompose a material sample into lattice cells. The lattice nodes can be considered as the centres of the lattice particles, which are connected by beams that can carry normal force, shear force, and bending moment. Cracks can be represented by simply removing the lattice particles. We can simulate the wave propagation in the samples by solving the equation of motion under an external force (Eq. 5). We adjust the external force so that the wavelength is greater than the length of the elements to ensure an accurate result^58^. Details of the equation of motion of the dLEM are referred to in a previous study^13^. The most relevant formulas for deriving dLEM are given below. In the dLEM, the resistance to axial, transverse and rotational displacements are modelled using three spring elements:

4 $$\begin{aligned} k_s = G\frac{A}{l}, \quad k_n = E\frac{A}{l}, \quad k_{\phi } = k_{n}\frac{l^2}{12}, \end{aligned}$$

where *A* is the cross-section area, *l* is the length of elements or Euclidean distance between nuclei, $k_s$ is the transverse stiffness, $k_n$ is the axial stiffness, $k_{\phi }$ is the rotational stiffness, *G* is the shear modulus and *E* is the Young’s modulus. These stiffness components are composed and transformed into the global coordinate system to obtain the global stiffness matrix. The general equation of motion under an external excitation is given in the following:

5 $$\begin{aligned} \varvec{M} \varvec{\ddot{u}}(t) + \varvec{C} \varvec{{\dot{u}}}(t) + \varvec{K} \varvec{u}(t) = f(t), \end{aligned}$$

where $\varvec{\ddot{u}}$, $\varvec{\dot{u}}$, $\varvec{u}$ represent accelerations, velocities, and displacements respectively, $\varvec{M}$ is the mass matrix, $\varvec{C}$ is the damping matrix, and $\varvec{K}$ the global stiffness matrix, *f* is an external excitation. The contact damping is neglected in the simulation. The mass matrix is assembled using the consistent mass matrix (CMM) by lumping the mass at the nodes. The global stiffness matrix is assembled by assuming a linear elasticity (according to Eq. 4).

The Newmark-$\beta$ method with the incremental formulation is adopted for solving the equation of motion.

6 $$\begin{aligned} \begin{aligned} \delta \varvec{\ddot{u}}&= \frac{1}{\beta \Delta t^{2}} \delta \varvec{u} - \frac{1}{\beta \Delta t} \varvec{\dot{u}} - \frac{1}{2\beta } \varvec{\ddot{u}}, \\ \delta \varvec{\dot{u}}&= \frac{\gamma }{\beta \Delta t} \delta \varvec{u} - \frac{\gamma }{\beta } \varvec{\dot{u}} + \Delta t (1 - \frac{\gamma }{2\beta } \varvec{\ddot{u}}), \end{aligned} \end{aligned}$$

7 $$\begin{aligned} \begin{aligned} \delta \varvec{\ddot{u}}&= \varvec{\ddot{u}}(t_{i+1}) - \varvec{\ddot{u}}(t_{i}), \\ \delta \varvec{\dot{u}}&= \varvec{\dot{u}}(t_{i+1}) - \varvec{\dot{u}}(t_{i}), \\ \delta \varvec{u}&= \varvec{u}(t_{i+1}) - \varvec{u}(t_{i}), \end{aligned} \end{aligned}$$

where $\beta$ and $\gamma$ are the Newmark-$\beta$ parameters; $\Delta t = t_{i+1} - t_{i}$; and $\delta \varvec{\ddot{u}}$, $\delta \varvec{\ddot{u}}$, $\delta \varvec{\dot{u}}$, and $\delta \varvec{u}$ are the increments of accelerations, velocities, and displacements. The incremental form of the Equation 5 becomes

8 $$\begin{aligned} \varvec{M} \delta \varvec{\ddot{u}} + \varvec{C} \delta \varvec{{\dot{u}}} + \varvec{K} \delta \varvec{u} = \delta f, \end{aligned}$$

By solving the motion equation, the displacements of the excited wave field are calculated for each time step. The wave displacements at specific locations are extracted as the observed wave data.

### Dataset generation

**Figure 1:**
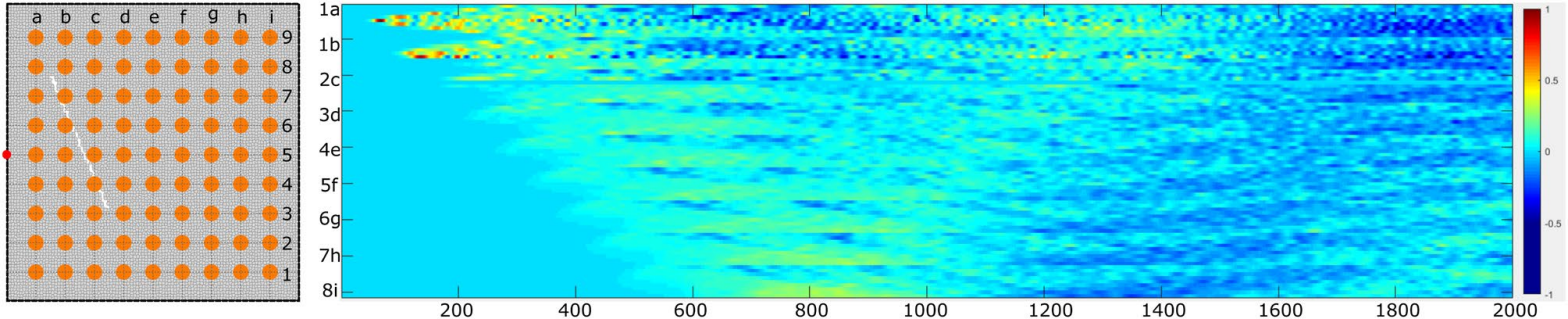
The placement of sensor array in sample domain and the recorded wave removal in the x-direction.

The dataset consists of homogeneous 2D plates with various cracks in terms of size, orientation, and location. Some samples containing no crack are generated as reference samples. The homogeneity means the lattice particles share the same density and Young’s Modulus. Generating the lattice particles involves a random drifting of regular grid nodes. Then, the nodes are used to generate Voronoi cells to represent the geometric shape of the lattice particles. To simulate damaged cases, randomly generated linear cracks are placed in the domain, and corresponding lattice particles are removed accordingly. Each sample contains $100\times 100$ lattice particles. The wave field is excited by a 1000*N* load in a short time span (in 3 time steps), and the displacements in the x- and y-direction are recorded for 2000 time steps. Figure 1 shows the setup for the virtual sensors and their recorded wave field history (in the x-direction). The red dot on the left middle edge indicates the location where the excitation is induced. The $9\times 9$ orange dots in the material domain are the locations of the sensors. The resulting wave field forms a $9\times 9\times 2000$-shaped array.

**Figure 2:**
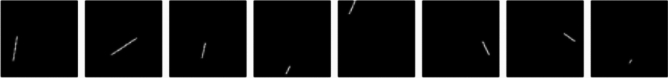
Examples of generated samples.

For each sample, a binarized image shows the ground-truth crack existence. The original image resolution is $100\times 100$, in alignment with the mesh size used by dLEM. The original image is then downsampled to the resolution required $16\times 16$ for prediction. The image pixels are set to 1 at the locations where the lattice nodes are removed to represent the presence of a crack. In contrast, 0 valued pixels indicate the intact material domain. The following figure (Fig. 2) shows some ground-truth images for the corresponding 2D plate samples.

### Dataset facts

**Figure 3:**
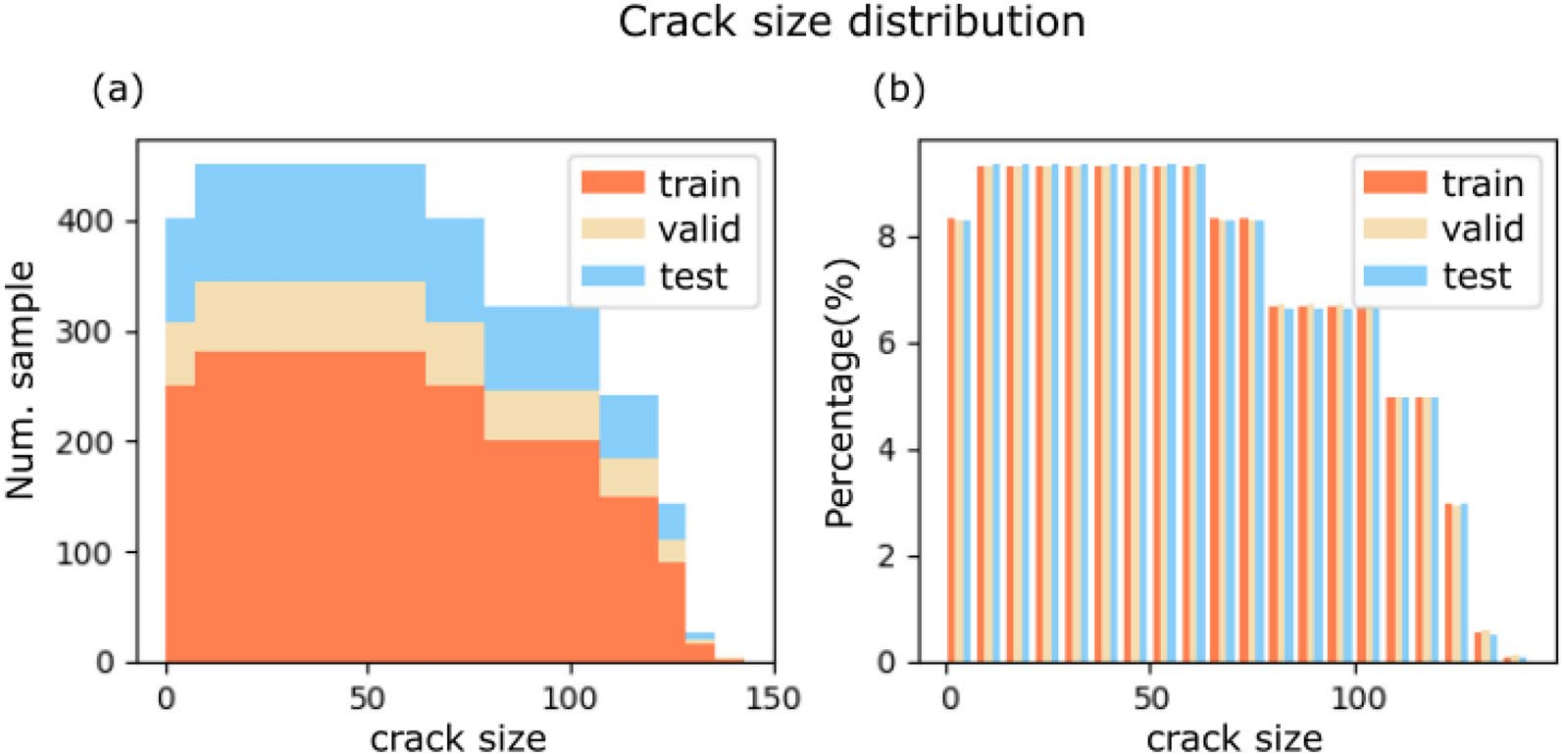
Distribution of crack size in the three sub-dataset (**a**) absolute number, (**b**) density.

The dataset consists of randomly generated samples in the same 2D domain, with a single, straight crack of random length, location, and orientation. A small portion of undamaged samples is also generated for the training dataset as references. The dataset has 4200 samples for training, 960 for validation, and 1600 for testing. An overview of the crack size distribution is given in Fig. 3. The crack size is specified as the number of damaged pixels among the ground-truth images’ total pixel numbers (100$\times$100). Stratified drafting ensures that the distributions of crack sizes among training, validation, and test datasets are similar.

### Preprocessing

Normalization is a crucial step to guarantee convergence and numerical stability since the value of the measured wave signal is very small. Previously, a scaling function for normalization was used. The min-max normalization function maps the input data X to the interval $[-1,1]\in R$. The normalized wave signals are then given to the neural network as input.

Besides a direct link between the measured wave signals and the crack’s existence, we developed an alternative preprocessing method using a reference wavefield. The idea is to learn from the difference between the wave signals measured from undamaged and damaged samples. We generated the reference wave signals as the average of the measured wave signals of all the undamaged samples in the training dataset. Then, the input becomes a normalized difference of the signals, subtracting the reference wave signals.

The insignificant change in the wavefield causes difficulty in detecting small cracks. By normalizing the difference, we scale the insignificant signals measured from samples with small cracks. We believe this preprocessing method helps the crack detection models to distinguish undamaged samples and samples with small cracks. Through empirical study, we can illustrate the efficiency of the preprocessing method.

## Deep neural networks for crack detection

In this work, the proposed models follow the encoder-decoder architecture^13,14^. Different backbone networks were tested for the encoder. Upsampling layers for the decoder were tested as well. We also developed a model with only an encoder followed by dense layers. It is also attractive since it uses fewer trainable parameters than all other models and achieves quite similar performance compared to other models. The proposed models are tested on the crack detection dataset to establish a benchmark.

**Figure 4:**
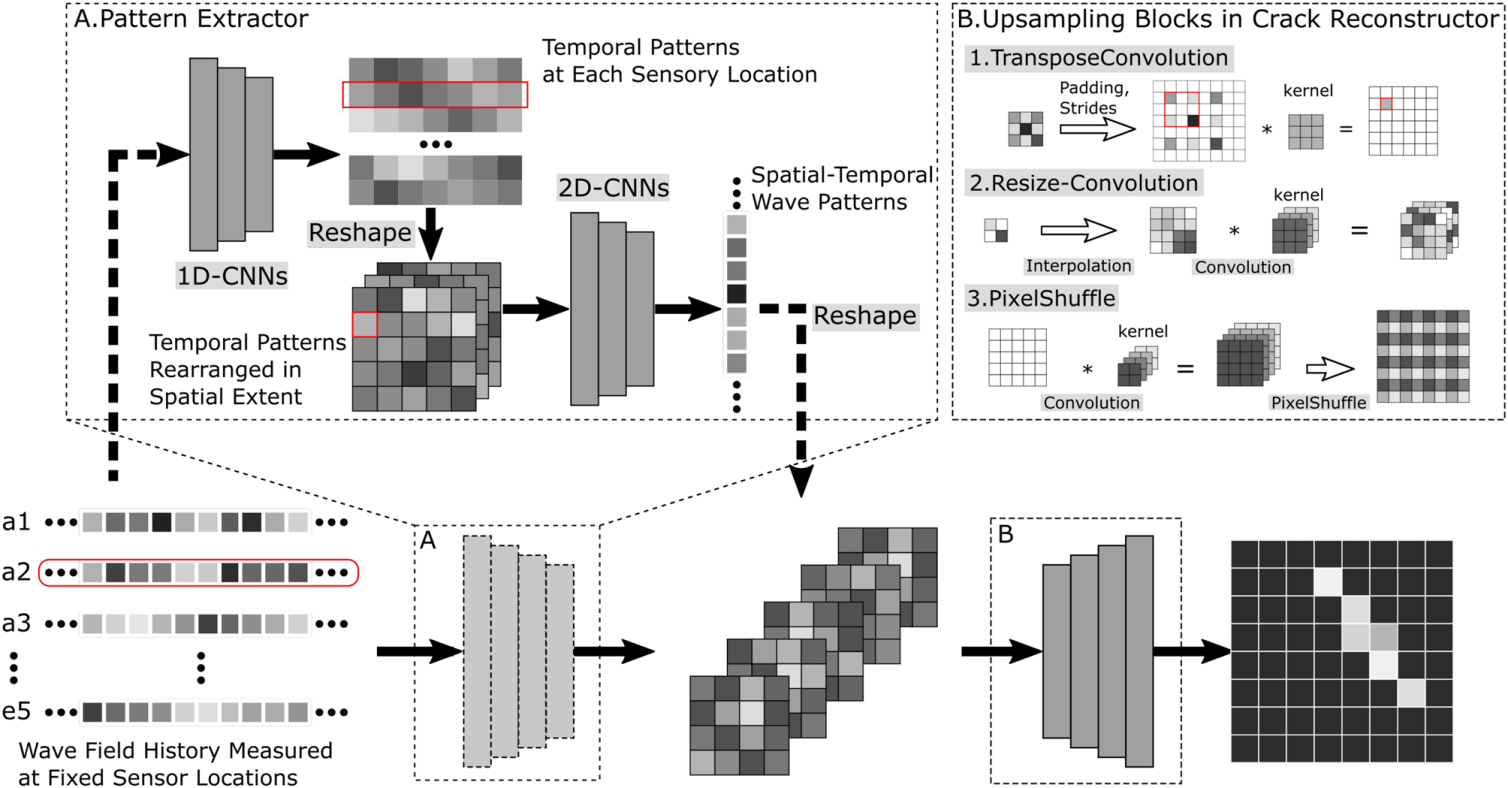
Schema of deep crack detection model.

Figure 4 illustrates a schematic drawing of the architecture. The encoder network extracts the spatiotemporal features from measured wave signals recorded at fixed sensor locations. We adopt the strategy first to handle the temporal and spatial dimensions. Thus, we implement the encoder network with a 1D version of backbone networks for extracting temporal information. Two 2D-CNN layers are followed to extract spatial information. Besides the previously tested networks, including the 1D version of stacked CNN (like VGG) and ResNet, we also tested a 1D version of DenseNet. The application of the 2D-convolutional layer for spatial information relies on the regular distribution of sensors. The decoder network reconstructs the probability of crack existence for the whole material domain. The primary operator in a decoder network is upsampling. The ideas of learnable upsampling layers come from computer vision tasks, such as image generation and segmentation. These layers include transpose convolution, resize-convolution, and pixel-shuffle. Additionally, we also discussed the possibility of making predictions without a decoder.

### The encoder network

**Figure 5:**
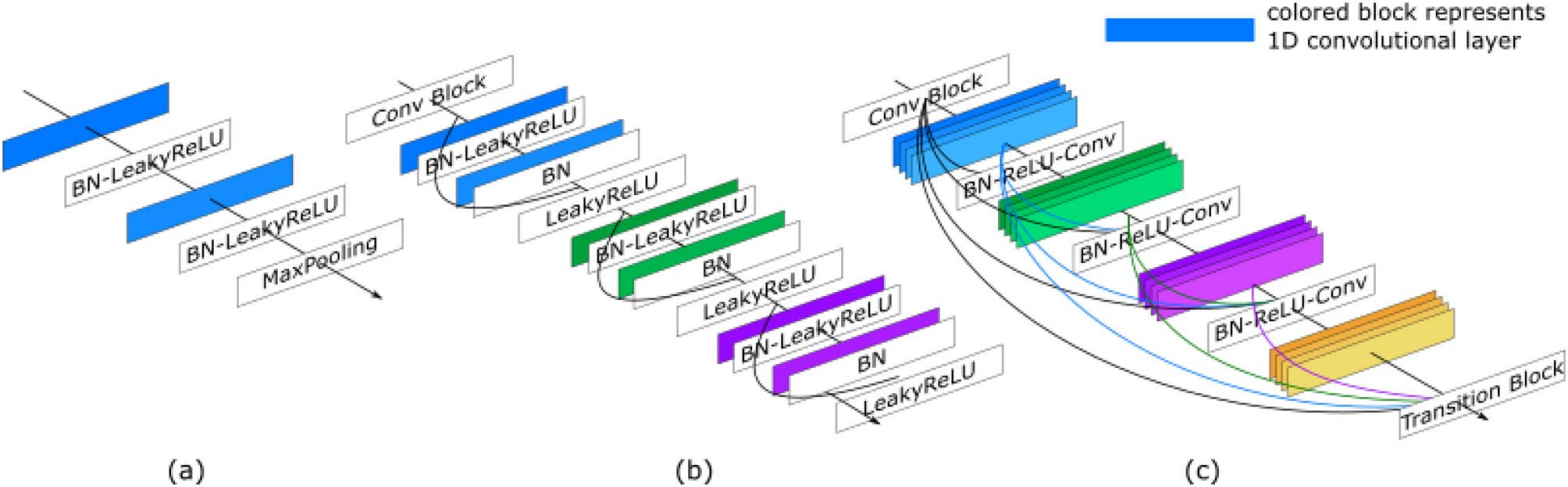
Tested backbone nets, (**a**) simply-stacked 1DCNN, (**b**) 1D-ResNet, (**c**) 1D-DenseNet.

The encoder network uses the 1D version of backbone networks for extracting temporal information. In Fig. 5, we show three basic blocks of the three backbone nets used for the encoder. The VGG-style network consists of stacked convolution layers followed by pooling layers to reduce the size of the temporal dimension. The ResNet and DenseNet employ skip connections to preserve the information from input data. Each backbone network consists of several such blocks accordingly. We want to point out that the 1D version of these networks can be implemented with 2D CNN, where the filter size is set to 1 along the sensory dimension to handle multiple sensors.

The VGG-style network consists of multiple convolutional layers. VGG-net proves that deeply stacked neural network layers can perform various computer vision tasks^51^. The previous study showed that such a stacked 1D-CNN could effectively extract spatial-temporal patterns from simulated wave signals^13^.

The following work showed that an advanced network, i.e. ResNet, facilitated better performance and reduced the required computational resource on the same dataset used for training the model with 1D-VGG-net^14^. ResNets are characterised by their skip connections to enable identity mapping and residual learning^52^. Using skip connections enables CNNs to be substantially deeper, more accurate, and more efficient to train. The residual block (ResBlock), consisting of two stacked convolution layers with non-linearity each, performs a mapping from input *x* to learned features *F*(*x*). Adding a skip connection changes the output feature to the following form,

9 $$\begin{aligned} H_l(x) = F_l(x)+x, \end{aligned}$$

where $F_l$ is the transformation of the $l^{th}$ residual block, and $H_l(x)$ is the corresponding output containing the transformed features and the identity of the input features.

DenseNet extends the idea of using skip connections by connecting each layer to every other layer in a feed-forward fashion. DenseNets has shown impressive results in many computer vision tasks, including image classification, semantic segmentation, and object detection. They have also been used in other domains, such as natural language processing and reinforcement learning^59–63^. We have modified the DenseNet with 1D convolutional layers, which are responsible for extracting the temporal features from wave signals, thus reducing the time dimension completely.

The batch normalization and activation functions are essential for these 1D version CNNs. Batch Normalization normalizes the prior layer outputs to a standard distribution, such that the distribution of output values remains while the weights of a layer are updated^64^. By reducing internal covariate shifts inside each batch, batch normalization stabilizes the training process and helps to accelerate the convergence of deep models.

The non-linearity Layer is also known as the activation layer. It performs a non-linear transformation on its input features. Many such functions exist, and the most relevant ones are discussed in this paper. Previous work showed that the Rectified Linear Unit (ReLU) could not work well in the backbone networks. It has a 0 gradient for negative input, i.e., the dying ReLu problem. LeakyReLU applies a parameter $0<\alpha <1$ for its negative input to guarantee a small gradient. It has been used for the 1D-VGG in previous work and performs well. In this work, we use a Scaled Exponential Linear Unit (SELU) that scales the Exponential Linear Unit (ELU) output with a scaling parameter $\lambda$^65^. ELU modifies ReLU by changing the constant 0 output for negative values to a weighted exponential function^66^. SELU is a self-normalizing activation function, meaning that it can maintain the mean and variance of activations close to a fixed point, which allows deep neural networks to be trained effectively.

**Figure 6:**
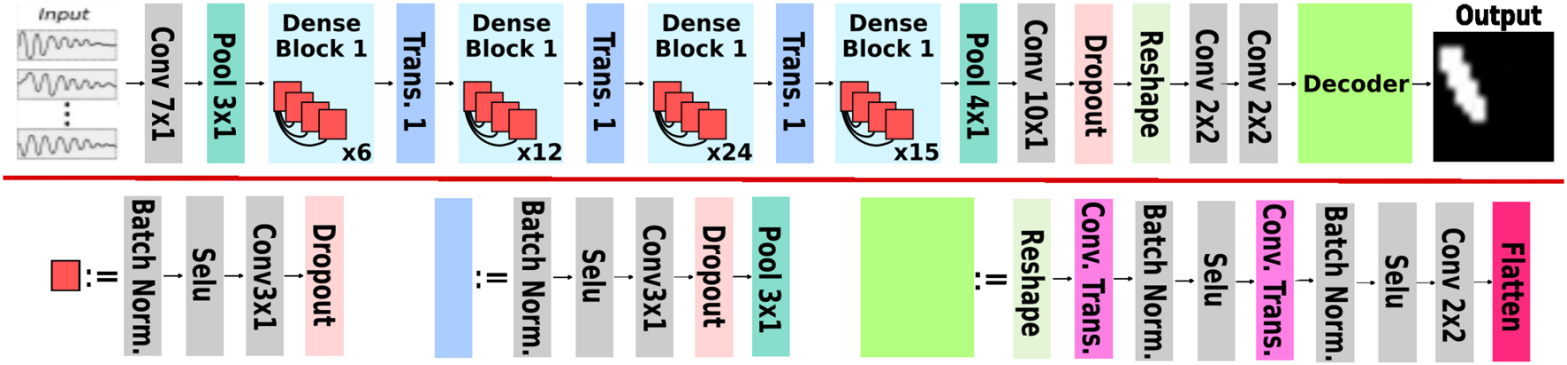
The crack detection network implemented with 1D-DenseCDNet as encoder.

Figure 6 shows a complete model implementation focusing on encoder that is mainly relied on 1D-DensNet. Four 1D Dense blocks are stacked to process the wave signals in the time domain. Then, the temporal features are reshaped according to their sensor locations and passed to two 2D convolution layers to learn spatial information, yielding a maximally condensed feature representation of $8\times 8$ spatial extent. Finally, the spatially rearranged features are passed to the decoder (marked in the green box) to predict crack existence at the desired spatial resolution.

### The decoder network

The decoder network consists of stacked upsampling layers to scale the input features to the desired spatial resolution. In this work, three types of trainable upsampling methods are evaluated. They are transposed convolution, resize and convolution, and pixel shuffle. The three upsampling methods are described in detail and also depicted in Fig. 4. Transposed convolution is also known as fractionally-stridden convolution or mistakenly as deconvolution. It rises from the need to use a transformation going in the opposite direction of a normal convolution, i.e., from the output space of convolution to its input space, while maintaining the connectivity pattern of the convolution^67^. The transposed convolution works by adding padding to expand the spatial dimension of input to compete with the spatial dimension of output and then performing convolution on the expended input. In transposed convolution, padding and stride are applied to output instead of input. Choosing stride equals 2; transposed convolution resizes the input with an up-scale factor of 2. Transposed convolutions suffer from the well-known Checkerboard Effect, i.e., the artefacts caused by uneven overlap at some parts of the features.

Pixel shuffle is an upsampling method used in super-resolution models to implement efficient sub-pixel convolutions with a stride of 1/*r*^68^. It rearranges elements in a tensor of shape $(H, W, C\times r^2)$ to a tensor of shape $(H \times r, W \times r, C)$. In detail, this operation treats the elements in the channel as sub-pixels and places these sub-pixels alternately in corresponding spatial locations. Usually, we need convolutional layer(s) before the pixel shuffle operation to transform the features into the desired number of channels.

Resize & convolution is proposed as an alternative to transpose convolution. The resizing layer upsamples the input feature map. The convolutional layer analyses the extracted features and prepares the spatial output. It is advantageous to avoid checkerboard artefacts^69^. Resize &convolution block consists of an interpolation layer (e.g., nearest-neighbour interpolation or bilinear interpolation) followed by a convolutional layer.

The encoder extracts the features of temporal and spatial dimensions and produces a feature map in the shape of (4,4,64). It is then forwarded to the decoder consisting of two upsampling blocks of the same type. Each of these blocks expands the spatial dimension of the input feature map. The first block is responsible for bringing the data into the shape of (8,8,64). The second block performs similar upsampling but produces an output in the shape of (16,16,16). The final convolutional layer produces the prediction in the shape of (16,16,1) with sigmoid activation. This is important because the output is expected to be between 0 and 1 as a possible crack existence.

## Benchmarking experiments

This section presents the benchmarking results for the neural network models on the crack detection task i.e. to predict the probability of crack existence in a sample. Firstly, we describe the loss function, metrics, and hyperparameters we employed for our experiments.

### The loss functions

The loss function measures the difference between the expected output and the actual output a neural network gives. It defines an optimization target for training the neural network. In image segmentation, loss functions are classified into distribution-based, region-based, boundary-based, and compound losses, i.e., a combination of several losses. Previous works employ focal loss, which belongs to distribution-based losses^70^. It is a variant of the binary cross-entropy loss that addresses the issue of class imbalance, where the target class consists of only a tiny portion of the image domain. It focuses more on the problematic cases and less consideration of the easy cases. Though proven powerful in previous studies, focal loss introduces two hyperparameters, which require unavoidable intensive experiments to find the optimal combination of the two parameters.

In this work, we use Dice loss. Dice loss is a region-based loss function, which is based on the concept of the Sørensen-Dice index^71^. The Sørensen-Dice index, known as the *Dice similarity coefficient* (DSC), is the most commonly used metric for evaluating accuracy in classification problems. The DSC is defined as the per voxel classification of true positives (TP), false positives (FP), and false negatives (FN):

10 $$\begin{aligned} \textrm{DSC} = \frac{2\textrm{TP}}{2\textrm{TP}+\textrm{FP}+\textrm{FN}}. \end{aligned}$$

Then, the Dice loss is defined as

11 $$\begin{aligned} {\mathscr {L}}_{\textrm{DSC}} = 1 - DSC. \end{aligned}$$

The Dice loss can handle class imbalance (to some degree) while keeping a simple form. The DSC value is always between 0 and 1, while its gradient is inherently unstable with highly class-imbalanced data where gradient calculations involve small denominators. We adopt the following form of Dice loss to reduce the risk of unstable,

12 $$\begin{aligned} {\mathscr {L}}_{\textrm{DSC}} = 1-\frac{2\textbf{I}+\varepsilon }{\textbf{U} +\varepsilon }, \end{aligned}$$

where $\textbf{I}= \sum _1^{N} y_i p_i$, $\textbf{U}= \sum _1^{N}( y_i + p_i )$, and $\epsilon$ is the smooth term.

We noticed that there are other possible loss functions, including *Tversky loss*, *Focal Tversky loss*, *Combo loss*, and so on. The Tversky index is modified from DSC by re-weighting the false positives and false negatives to address the imbalance issue. Focal Tversky loss modifies the Tversky loss by adding the focal parameters. The Combo loss is an example of compound loss, which sums up the Dice and cross-entropy loss. Since a review of loss functions is beyond the scope of this paper, we refer to the detailed review article^72^.

### Metrics

We follow the metrics often used in semantic segmentation to evaluate the models’ performance. *Precision* is the portion of correctly identified damage (TP) among all predicted damage (TP+FP), while *recall* is the portion of correctly identified damage (TP) among all actual damages (TP+FN). The crack detection task is a significant class imbalance problem, i.e., many more undamaged regions than damaged regions in each sample. Precision and recall are inappropriate metrics if we consider only damaged and undamaged classes since the model can trivially achieve high performance when most (or all) pixels are labelled as undamaged. In this case, we must use them to quantify a model’s performance. The harmonic mean of precision and recall (Eq. 13) is called the F-1 score, which is equivalent to the Dice coefficient (DSC).

13 $$\begin{aligned} F = \frac{2\cdot precision\cdot recall}{precision+recall} \end{aligned}$$

Besides DSC, *Intersection over Union* (IoU) metric is another commonly used metric for semantic segmentation tasks. The IoU metric is closely related to the Dice coefficient. It provides a geometric interpretation for the performance by quantifying the percentage overlap between the true damaged area and the predicted damaged area. The IoU metric is computed by the following equation (Eq. 14). Compared to the Dice coefficient, IoU is more sensitive to the change of FP and FN, while both metrics agree on the evaluation. If one model performs well under one metric, it is also evaluated as working well under another.

14 $$\begin{aligned} \textrm{IoU} = \frac{\textrm{TP}}{\textrm{TP}+\textrm{FP}+\textrm{FN}}. \end{aligned}$$

The correctness is defined based on the IoU value as its predicted IoU is more significant than a given threshold. The output of the models can be interpreted as confidence (probability) of crack existence. Given the threshold, this soft prediction can be transformed into a binary one. The accuracy of the whole dataset is calculated as the ratio of the number of correctly predicted cases to the total number of evaluated datasets.

### Settings and hyperparameters

The Adam optimizer is a commonly used algorithm for training deep neural networks^73^. We employed it in our experiments with its default learning rate of 0.001 in all experiments. Each training lasts for 200 epochs, and we chose the best performing model *w.r.t.* DSC on the validation data.

*Dropout* is a widely used regularization technique to prevent overfitting in deep neural networks^74,75^. Its main idea is to drop “neurons” randomly and their connections during training to prevent complex co-adaptations on training data. We randomly “drop” all features from the same sensor, resulting in an “average” of models generated by the random drop strategy. In experiments, we set the dropout rate to 0.8, requiring ca. 65 out of 81 sensors not to be used during training.

### Results

We adopt the same activation function (SELU) and dropout rate (0.8) across all models to compare different encoders and decoders. This can be particularly useful when isolating the impact of the architectural differences between models. We modified the previous models by switching LeakyReLU to SELU, where 1D-CNN and 1D-ResNet are used as the encoder and trained on the new dataset. The 1D-DenseNet encoder with different decoders and without a decoder are also developed and tested.

First, it’s necessary to explain that we have binarized predictions at the pixel level. The chosen threshold (binarising threshold) is 0.5, meaning that a prediction value higher than 0.5 for each pixel indicates the possible presence of a crack. Whether the prediction of a crack is correct is defined through the IoU value. This means accurately predicted crack pixels should exceed a certain threshold for the total number of crack pixels. We have selected an IoU threshold 0.5 as the accuracy threshold (or IoU threshold).

**Figure 7:**
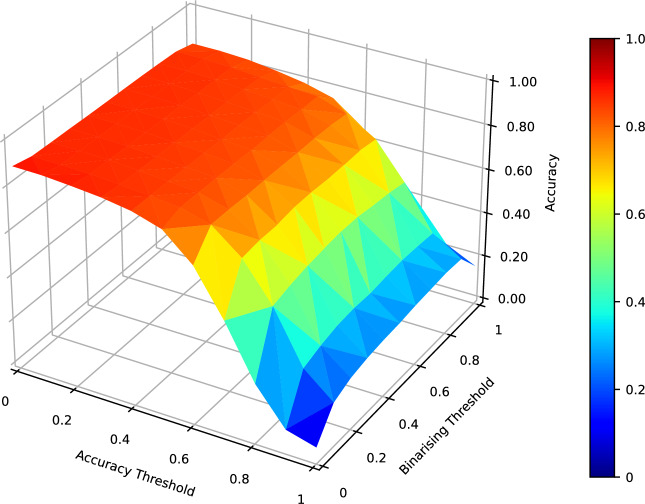
Accuracy influenced by the choices of IoU threshold and binarising threshold (taken from the evaluation of model 1D-ResNet-Resize &Conv ).

**Figure 8:**
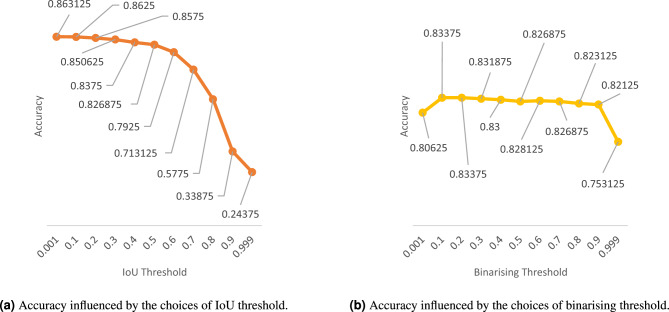
Accuracy influenced by (**a**) IoU threshold when binarising threshold is 0.5, and (**b**) binarising threshold when IoU threshold is 0.5 (taken from the evaluation of model 1D-ResNet-Resize &Conv ).

To illustrate the rationale behind the threshold selection, let’s take the model with 1D-ResNet as the encoder as an example. In Fig. 7, we illustrate the relationship between different thresholds and the final accuracy. If we demand that every pixel be accurately classified to represent correct crack identification (IoU$\rightarrow$1), the accuracy will drop sharply. Though this could ensure absolute accuracy, it’s challenging to achieve in practice. Considering a very low threshold (IoU$\rightarrow$0), even if a very small number of damaged pixels are identified, it could be considered a correct crack identification. However, this ignores many falsely identified pixels, which is also impractical. We found that around the threshold of 0.5, we can ensure a certain number of correctly identified pixels while maintaining a reasonable accuracy level. Figure 8 further illustrates the impact of different IoU thresholds and different binarizing thresholds on accuracy when the binarizing threshold is set to 0.5 and when the IoU threshold is set to 0.5. Regarding the binarizing threshold, accuracy is minimally affected except for extreme values (close to 0 or 1). This indicates that the model’s pixel-level predictions have a high level of confidence.

**Table 1:** Evaluated metrics for the benchmarking models.

	Accu	IoU	DSC	Prec	Recall
1D-CNN-Resize &Conv	0.81875	0.756758	0.861539	0.869824	0.853411
1D-ResNet-Resize &Conv	0.826875	**0.772105**	**0.871399**	**0.883311**	**0.859803**
1D-DenseNet-no decoder	0.803125	0.717579	0.83557	0.83317	0.837984
1D-DenseNet-TConv	**0.836875**	0.76014	0.863727	0.875395	0.852366
1D-DenseNet-Subpixel	0.83625	0.757815	0.862224	0.880307	0.844868
1D-DenseNet-Resize &Conv	0.834375	*0.768578*	*0.869148*	*0.881086*	*0.857529*

The best values are in bold.

**Table 2:** Evaluated metrics for the normalization method with reference wave field.

	Accu	IoU	DSC	Prec	Recall
1D-CNN-Resize &Conv	0.8675	0.760874	0.8642	0.836427	0.893882
1D-ResNet-Resize &Conv	0.884375	0.767203	0.868268	0.841303	**0.897019**
1D-DenseNet-Resize &Conv	**0.88625**	**0.7856**	**0.879929**	**0.877088**	0.882788

The best values are in bold.

Table 1 presents all metrics on the selected model, including IoU, Dice Similarity Coefficient (DSC), Precision, Recall, and accuracy. Accuracy is calculated based on whether each damaged region (pixel) is predicted accurately above 50%. IoU, DSC, Precision, and Recall are calculated by assessing whether each region (pixel) is accurately classified (has a crack or no crack). Networks based on encoder-decoder architecture perform better in crack detection. For the decoder network, whether using a VGG-like CNN or a more complex network, such as ResNet and DenseNet, relevant features associated with cracks can be extracted from wave field signals.

More advanced networks like DenseNet exhibit superior performance regarding crack prediction accuracy, while ResNet performs better in pixel-wise predictions. When employing different decoder networks, conventional Transpose Convolution yields satisfactory results. However, Resize & Convolution demonstrate advantages in pixel-level predictions. We speculate that this is due to the limited impact of the chessboard effect on the whole crack detection but a larger influence on pixel-level predictions.

It’s worth noting that even though we included a model with an upsampling decoder, it can be understood as employing fully connected layers for decoding. While this approach omits an upsampling decoder network and still achieves around 80% accuracy, its performance remains weaker than the upsampling version. Another drawback of using fully connected layers for decoding is the challenge of expanding spatial resolution. For predictions at higher resolutions, such as 32$\times$32 or 64$\times$64, the parameters that must be trained for the fully connected layers will grow exponentially.

**Figure 9:**
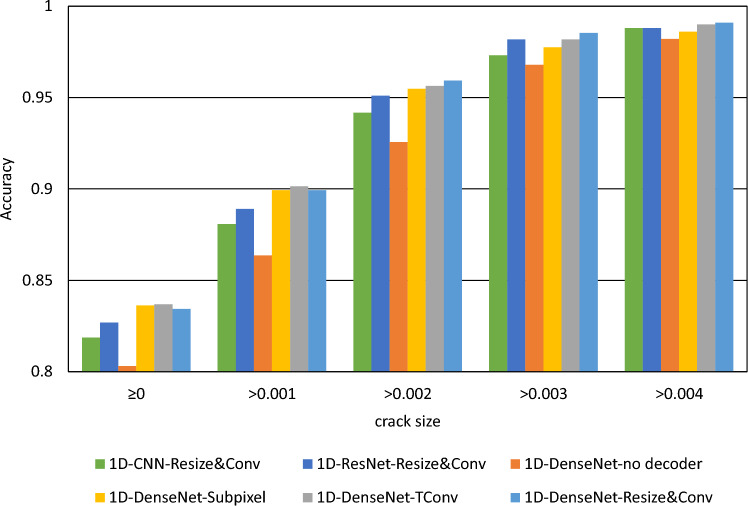
The prediction accuracy of tested models evaluated across various crack size groups.

In general, using a more powerful encoder network can better extract features from wave field signals relevant to cracks. However, the selection of the encoder network regarding the scale and complexity still requires careful consideration. Larger networks have more trainable parameters, which demand more data and robust training methods. Additionally, larger networks have the risk of overfitting when training data is limited. On the other hand, by comparing the performance of models on cracks of different sizes, we found that predicting smaller cracks is more challenging than larger ones. In Fig. 9, the accuracies of the models trained with the unnormalized dataset are shown. To show the influence of crack size to the performance, the results for the whole test set as well as for the adjusted test set of excluding smaller cracks are given. For larger cracks, the differences in the performance (e.g., accuracy) among different models (including various encoder networks and no decoder versions) are minimal. In contrast, for smaller cracks, more advanced networks tend to produce better results for predicting cracks. We attribute this to the fact that the wavefield changes caused by smaller cracks are very insignificant, making them difficult to capture by sensors and more susceptible to irregularities in particle shapes and distributions within the material and measurement noise. This consideration also prompts us to contemplate amplifying such wavelength variations in the data.

**Figure 10:**
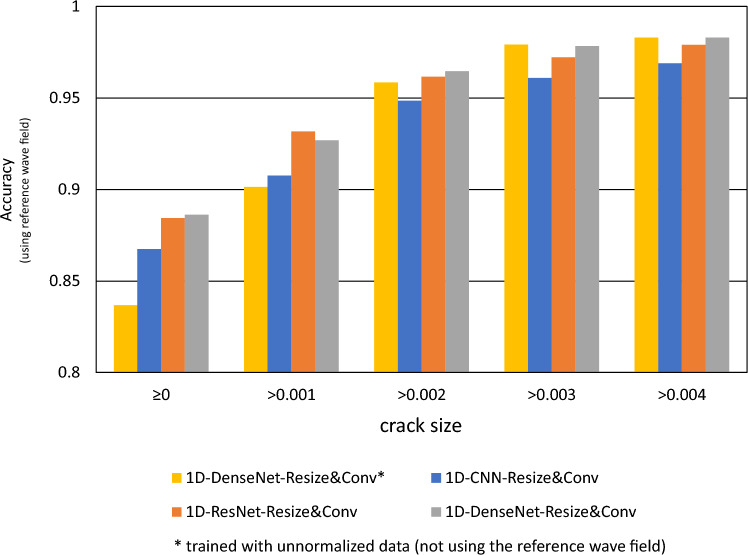
The prediction accuracies assessed across different crack size groups for the models trained with with reference wavefield.

The evaluation of training the models with the normalization method is shown in Table 2. It presents all metrics on the selected model trained using a reference wave field. The reference wave field is an average wave field from different intact samples. We trained each encoder network with five models and took the median value of their metrics. We have discovered that training with reference wavefields significantly improves the models employing different encoder networks. Moreover, more advanced encoder networks, such as 1D-DenseNet, appear to benefit more from this approach. Figure 10 illustrates the accuracies of the models trained with normalized dataset over different crack sizes. The model of 1D-DenseNet with resize and convolutional upsampling method is chosen as the reference model (marked as “*” in the Figure). It is observable that the 1D-CNN model with the Resize & convolution method trained with normalized data out performs the model of 1D-DenseNet and the same upsampling method trained with unnormalized data. It even outperforms other models trained without using the reference wavefield, showing the advantage of using the normalization method. We can aslo observe improved accuracy when we exclude the test samples with smaller cracks. This agrees with the previous finding that smaller cracks are more difficult to detect. The most important is using a reference wavefield in normalization improves the performance largely on the samples with smaller cracks.

## Conclusion and future work

Numerical simulation is a cost-efficient way of creating vast data for training deep neural networks. It is a flexible way of producing data in different damage scenarios, rarely seen in a real-world structure. This work shows an upgraded dataset for the crack detection problem generated by running many numerical simulations. We noticed difficulties detecting small cracks, so we kept a similar distribution of crack sizes in the training, validation, and testing datasets.

We defined crack detection for plates as a semantic segmentation problem. We proposed a general neural network architecture employing an encoder and a decoder. The encoder and decoder’s backbone networks can be implemented as well-developed neural networks, such as ResNet and DenseNet for the encoder and transposed convolution for the decoder. The backbone of encoder networks is modified to the 1D version for measured wave signal data. The decoder consists of stacked upsampling layers to scale the spatial-temporal feature map to a desired spatial resolution. Though it is possible to predict cracks at a coarse spatial resolution, upsampling layers facilitate prediction at a finer resolution. We generated a dataset to benchmark the variations of architecture and identified the main influence factors of the model’s performance. Variations of the model’s components have been tested, including different encoder backbone networks (VGG, ResNet, DenseNet) and upsampling blocks (transposed convolution block, sub-pixel block, and resize-convolution block). We identified that normalizing the input data using reference wave signals significantly increases the model’s performance for small cracks. We point out that this method inherently scales the modified wave signals of small cracks. The paper reaches its limits in detecting extremely small microcracks caused by two main challenges. First, the model is designed for cracks of different sizes, which makes precise control harder and is not specific to a single crack group or size. Secondly, the weak signals associated with small cracks could challenge the model in this crack category, as the waves can be easily disturbed in the case of more minor cracks. A possible solution to this problem could be to adjust the data processing or modify the complexity of the model, perhaps by combining multiple models to consider different crack groups.

Future work could include a two-fold improvement of the model’s performance by applying more powerful components and focusing on small cracks. More neural networks would be tested for spatial-temporal feature extraction. For example, the network that handles sequential data, such as RNNs, LSTM, and attention blocks, can extract temporal patterns. It is also possible to fine-tune a well-trained model on a dataset that consists of small cracks. We also noted that our proposed method relies on predefined sample dimensions and shapes. Future extension of our method from these two aspects is needed, i.e., extending from 2D plate to 3D samples and considering different sample shapes. The ultimate goal of our research is to provide knowledge that helps to build a real-time, cost-efficient diagnosing system for civil structures.

## Data Availability

The datasets generated during and/or analysed during the current study are available from the corresponding author on reasonable request.
